# Supplementary material for: High-quality haploid genomes corroborate 29 chromosomes and highly conserved synteny of genes in Hyles hawkmoths (Lepidoptera: Sphingidae)
Source: BMC Genomics. 2023 Aug 7;24:443. doi: 10.1186/s12864-023-09506-y (PMC10405479; doi:10.1186/s12864-023-09506-y)
Supplement: Supplementary file 3 — Additional file 3: Table S2. Accession numbers of reference wing pattern sequences and data type used for blat in the genome browser. Manduca sexta WntA and optix are not displayed on the NCBI web portal. [file 12864_2023_9506_MOESM3_ESM.docx]

**Table S2**

**Accession numbers of reference wing pattern sequences and data type used for blat in the genome browser.** *Manduca sexta* *WntA* and *optix* are not displayed on the NCBI web portal.

| **Gene** | **Reference species** | **Accession number** | **BLAT** |
| --- | --- | --- | --- |
| *WntA* | *Heliconius himera* | AFC75686.1 | Protein sequence |
| *optix* | *Heliconius erato* | KC469894.1 | Protein sequence |
| *cortex* (isoform X1) | *Manduca sexta* | XM_030179809 | mRNA sequence |
| *aristaless* | *Manduca sexta* | XM_030185306.2, XM_037442080.1- XM_037442082.1 | mRNA sequences |
| *Distal-less* | *Manduca sexta* | AY616435.1 | mRNA sequence |
|  |  |  |  |
